# Improved Detection and Fragmentation of Disulphide-Linked Peptides

**DOI:** 10.3390/mps1030033

**Published:** 2018-09-03

**Authors:** Evelyne Maes, Stefan Clerens, Jolon M. Dyer, Santanu Deb-Choudhury

**Affiliations:** 1 Food & Bio-Based Products, AgResearch Ltd., 1365 Springs Road, Lincoln 7674, New Zealand; stefan.clerens@agresearch.co.nz (S.C.); Jolon.dyer@agresearch.co.nz (J.M.D.); Santanu.deb-choudhury@agresearch.co.nz (S.D.-C.); 2 Biomolecular Interaction Centre, University of Canterbury, Christchurch 8140, New Zealand; 3 Riddet Institute, Massey University, Palmerston North 4442, New Zealand; 4 Wine, Food & Molecular Biosciences, Lincoln University, Lincoln 7674, New Zealand

**Keywords:** disulphide bonds, MALDI-TOF/TOF MS, aniline, proof-of-existence

## Abstract

Characterisation of peptides containing intact disulphide bonds (DSBs) via mass spectrometry is challenging. Our study demonstrates that the addition of aniline to alpha-cyano-4-hydroxycinnamic acid improves detection and fragmentation of complex DSB peptides by matrix-assisted laser desorption/ionization, tandem time-of-flight mass spectrometry (MALDI-TOF-TOF MS). This improved assignment will be a significant new tool when a simple screening to confirm the DSB existence is required.

## 1. Introduction

Disulphide linkages between the thiol groups of two cysteine residues are a key post-translational modification in proteins and are highly important to the stability of protein structures. Mapping these disulphide bonds (DSBs) and determining cysteine residue pairing is therefore crucial to protein characterisation. As proteins are often used as therapeutics, many different procedures to check disulphide bond arrangement are included in quality control procedures of therapeutic protein production, ensuring protein safety and efficacy [1]. Typically, systematic disulphide analysis by mass spectrometry includes a two-step process, wherein first, the number of DSB and number of free cysteines is determined, followed by the probability and mapping of disulphide connectivity [1]. Depending on the protein, a range of different strategies might need to be applied, including no DSB cleavage all the way to gas phase DSB cleavages [2].

Matrix-assisted laser desorption/ionization, tandem time-of-flight mass spectrometry (MALDI TOF-TOF MS), is frequently used for the analysis and characterisation of peptides and proteins. Compared to electrospray ionisation (ESI), MALDI has two major advantages which can help interpretation of disulphide linkages: (1) MALDI produces mainly singly charged ions, which allows less complex mass spectra; and (2) MALDI produces intact peptide ions without excessive fragmentation. Applying MALDI-MS to cross-linked peptides adds another level of complexity. This mainly because, the overall detection of cross-linked peptides in an MS spectrum is challenging as their abundance is low compared to their linear counterparts. Consequently, isolating and fragmenting the peptide-of-interest’s corresponding mass-to-charge (*m*/*z*) ratio becomes crucially important to characterising and confirming the putatively disulphide linked peptides [3].

The aim of this work is to obtain a method that allows a more efficient detection and fragmentation of peptides containing DSBs in non-reduced proteins. We report how incorporating aniline in a common MALDI matrix, alpha-cyano-4-hydroxycinnamic acid, improves both DSB detection in MS as well as DSB peptide fragmentation behaviour compared to other additives and matrices. Although the use of aniline as a MALDI matrix additive has been successfully used to increase signal-to-noise ratios for peptide profiling in tissue imaging [4], improve glycan detection [5] and has been applied in lipid research [4], we describe for the first time the benefit of this additive to DSB analysis. 

## 2. Materials and Methods

### 2.1. Materials

Lysozyme C (from chicken egg white), bovine beta-lactoglobulin and aniline (ACS reagent grade, ≥99.5%) were purchased from Sigma–Aldrich (St. Louis, MO, USA). Sequencing grade modified trypsin was purchased from Promega (Madison, WI, USA). Acetonitrile, acetone, and ethanol were all liquid chromatography–mass spectrometry (LC-MS) grade solvents from Fluka Analytical, by Merck (Kenilworth, NJ, USA). Optima LC-MS grade water and trifluoroacetic acid were obtained from Fisher Scientific (Hampton, NH, USA). Ammonium acetate was purchased from VWR (Radnor, PA, USA), Tri-sodium citrate from BDH laboratories (Dubai, UAE). Alpha-cyano-4-hydroxycinnamic acid (HCCA), 2,5-dihydroxyacetophenone (DHAP), bovine serum albumin (BSA) digest (500 fmol/µL), and peptide calibration standard were purchased from Bruker Daltonics (Bremen, Germany).

### 2.2. Sample Preparation

One milligram of Lysozyme C protein was dissolved in 500 µL of 0.1 M ammonium bicarbonate buffer. 1.5 mg of beta-lactoglobin was dissolved in 750 µL of 0.1 M ammonium bicarbonate and a Bruker BSA digest (5000 fmol) aliquot was dissolved in 10 µL 0.1% TFA.

To start the tryptic digestion of each of the proteins, 2 µg of trypsin (20 µg dissolved in 40 µL 0.1 M ammonium bicarbonate) was added to 100 µg of protein, the sample was vortexed and incubated overnight at 37 °C with vortexing at 300 rpm (Thermomixer, Eppendorf (Hamburg, Germany). After 16 h, the digest was stored in −80 °C until further use. Before MALDI-MS analysis, the samples were diluted 1:1 in 0.1% TFA.

### 2.3. MALDI Matrix Preparation

The HCCA matrix was prepared by a 10-fold dilution of a saturated HCCA matrix solution in acetone using ethanol:acetone:0.1% TFA (ratio 6:3:1, v:v:v). The DHAP matrix solution was prepared by dissolving 7.6 mg of DHAP in 500 µL ethanol. The mixture was vortexed for 1 min followed by a 15 min sonication. For both matrices, 4 separate 0.5 mL safe lock tubes were prepared. One of them contained matrix only, and the other three had matrix plus one of the following additives: 10 mM ammonium acetate, 10 mM tri-sodium citrate or aniline (10 µL neat aniline in 100 µL matrix; (M:A 10:1)) [6,7,8]. Different ratios of HCCA:aniline were prepared and represent the mixture in matrix (M):additive (A), v:v.

To ensure equal mixing, 1 µL of sample and 1 µL of matrix solution were spotted onto parafilm. After mixing, 1 µL of solution was spotted onto an Anchorchip MALDI target plate.

### 2.4. Mass Spectrometric Measurements

Dried sample droplets were analysed using an Ultraflex III MALDI-TOF/TOF MS (Bruker Daltonics) in positive ion reflectron mode with an *m*/*z* range of 400–5000 Thomson and equipped with a 200 Hz Nd:YAG laser. Laser intensity was optimized for each sample. Spectra were recorded from the sum of at least 2500 laser shots. To reduce fluctuations in ionisation efficiency, this sum of shots is the result of different positions within each spot. For every type of matrix, spectra were externally calibrated with the Peptide calibration standard (Bruker Daltonics). Post-acquisition processing of data was performed utilizing FlexAnalysis, version 3.4 software (Bruker Daltonics).

## 3. Results and Discussion

The goal of this study was to develop a MALDI-TOF/TOF MS method that improves the efficiency of detection and fragmentation of peptides containing intact disulphide bonds while keeping the lab-time minimal. To achieve this, we compared the ability to detect and fragment disulphide-linked peptides between a very commonly used MALDI matrix in peptide research, HCCA and the matrix DHAP. This latter matrix has previously been described as a primary choice when measuring peptides containing free SH-groups (or alternatively cysteine-containing peptides). These two matrices (m) were tested in combination with three different additives: ammonium acetate (aa), sodium citrate (sc), and aniline (ani). We chose to use a non-reduced protein digest of Lysozyme C as a model for intact disulphide bonds. We allowed disulphide scrambling to increase the number of potential DSB peptides by not alkylating the free cysteines before tryptic digestion, where scrambling is favoured by the proteins’ environment (pH 8.5 and 37 °C).

### 3.1. Effect of Different Matrices and Additives on Peptide Calibrant Signal

**Figure 1:**
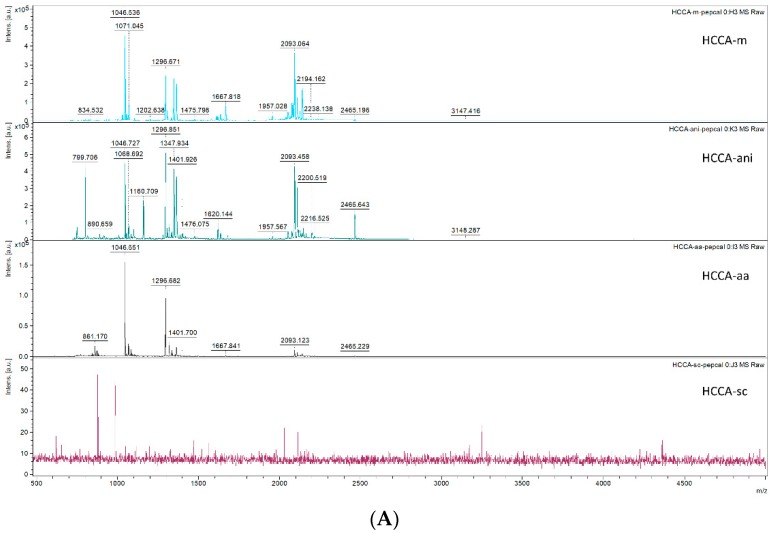

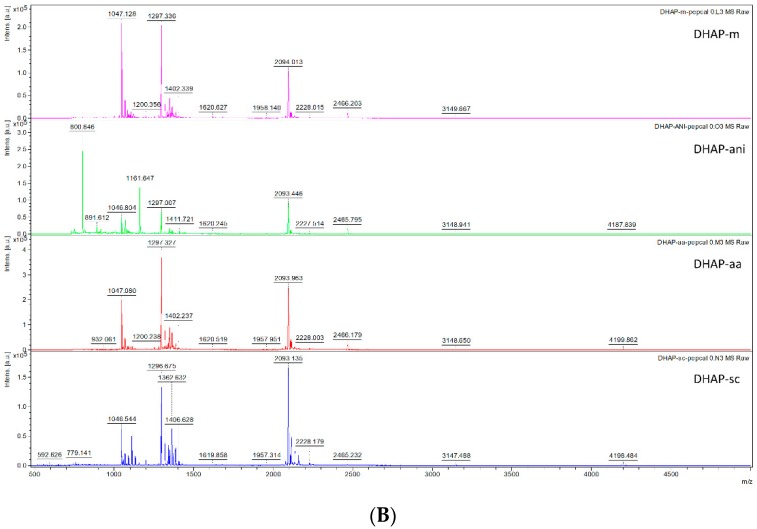
Matrix-assisted laser desorption/ionisation (MALDI) mass spectra of the Bruker peptide calibration mix. X-axis represent mass-to-charge (*m*/*z*) and y-axis represent signal intensities in arbitrary units [a.u.]. (**A**) Mass spectra in Alpha-cyano-4-hydroxycinnamic acid (HCCA) matrix (m) and three different additives ammonium acetate (aa), sodium citrate (sc) and aniline (ani). (**B**) MS spectra of 2,5-Dihydroxyacetophenone (DHAP) matrix (m) and the three different matrix additives (aa, sc, and ani).

To evaluate the overall linear peptide ionisation efficiency of the different matrix:additive (M:A) combinations, we analysed the Bruker peptide calibrant mixture using different M:A combinations on separate MALDI target spots and measured a sum of 2500 shots taken from different regions of the target spot. The Bruker Calibration standard II contains eight peptides: Bradykinin 1–7 (757.399 Da), Angiotensin II (1046.541 Da), Angiotensin I (1296.684 Da), Substance P (1347.735), Bombesin (1619.822 Da), ACTH clip 1–17 (2093.086 Da), ACTH clip 18–39 (2465.198 Da) and Somatostatin 28 (3147.471 Da). Upon comparison of the M:A combinations, the HCCA-sc matrix was not successful in detecting any peaks of the peptide calibrant solution. In Some M:A combinations one peak of the eight peptide calibrant mixture was presumably below the detection limit: Bradykinin was missed by HCCA-ani, DHAP-m, DHAP-aa and DHAP-ani. Bombesin was missed by HCCA-aa. (Figure 1A,B) Comparison of the signal intensities of each peptide across the different M:A options does not have a clear favourite outcome, although the peptide signal intensity (as well as signal-to-noise, S/N) was generally higher in HCCA-m or HCCA-ani Table 1). Interestingly, somatostatin 28, detected in most M:A combinations, though in low intensity, contains an intra-peptide disulphide bond and can be used as a guiding peptide for our approach.

### 3.2. Effect of Different Matrices and Additives in Detecting Disulphide Linked Peptides

**Figure 2:**
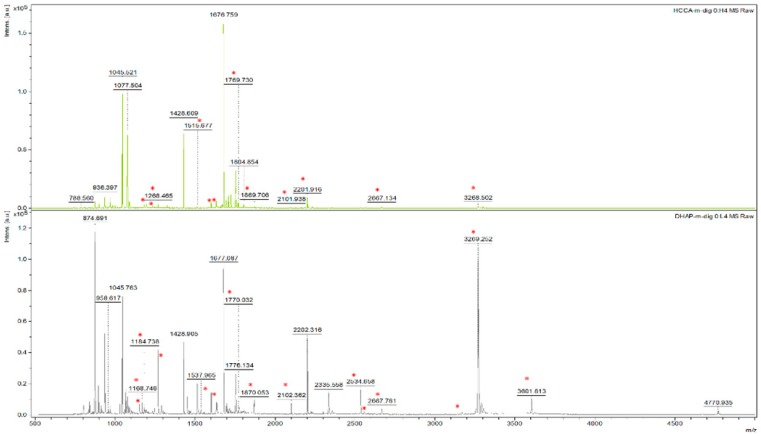
MS spectrum of non-reduced Lysozyme C digest in HCCA (upper panel) or DHAP (lower panel) matrix. The red dots indicate potential disulphide-linked peptides (DSB).

To study the ability of detecting disulphide bonds with different M:A combinations, we selected a tryptic digest of chicken Lysozyme C as an example. The protein contains 8 cysteine residues, has 4 essential DSB for proper protein folding, but can form up to 21 potential DSBs (when single disulphide bonds are formed between tryptic peptides within the 750–5000 Da mass range) if disulphide scrambling of the tryptic digest is allowed. Detecting these cross-linked peptides was done by preparing a non-reduced protein digest and measuring it in combination with the different matrix options. The masses of the linked peptides were calculated by the following rule: a disulphide-linked peptide, A-S-S-B, with A and B different peptides and S-S being a disulphide cross-link, is detected at an *m*/*z* ratio that equals the sum of the *m*/*z* ratios of both peptides minus the mass of two hydrogen atoms plus one proton. An overview of potential DSBs detected with the different M:A combinations is represented in Table 2. Similar to the calibrant peptide mix, HCCA-sc was unable to produce an MS spectrum of good quality. Overall, more potential DSB peptides could be detected with DHAP matrix compared to HCCA matrix (Figure 2, red dots).

### 3.3. Ability to Isolate and Fragment Potential Disulphide Bonds

To confirm and prove that the putative disulphide bonded peptides do contain a DSB, the matching *m*/*z* precursors need to be isolated and fragmented. Although more potential matching masses were detected in the MS spectrum in the DHAP matrix combinations, HCCA clearly outperformed the matrix in terms of fragmentation ability (see the great amount of green highlighted squares in HCCA than in DHAP combinations in Table 2). Despite the HCCA matrix performing quite well alone, the addition of aniline seems to provide extra fragmentation ions that lead to additional sequence information of DSB peptides. Specifically, aniline improves the characterisation of more complex linkages. While putative disulphide bonded peptides were detected by both HCCA-m and HCCA-ani, some could only be fragmented with HCCA-ani. 

### 3.4. Optimising the HCCA Matrix:Aniline Ratio for Disulphide Bond Fragmentation

Next, we optimized the ratio of HCCA matrix:aniline to check with ratio has the best DSB detection, isolation and fragmentation ability. With the previous HCCA matrix:aniline ratio being 10:1, v:v, we opt to test also 100:1, 5:1, 1:1 and 1:5 ratios. These different mixtures were tested with both somatostatin 28 and Lysozyme C digests and every measurement was performed in triplicate. Supplementary Figure S1A displays the signal intensities of Somatostatin 28 in these different matrix:additive ratios. In supplementary Figure S1B, the HCCA-matrix:aniline ratio comparison for the Lysozyme C digest is displayed. In both cases, the 10:1 ratio seems to give the best result and is therefore used in the rest of this work.

### 3.5. Characterisation of Inter-Peptide Disulphide Bonds

**Figure 3:**
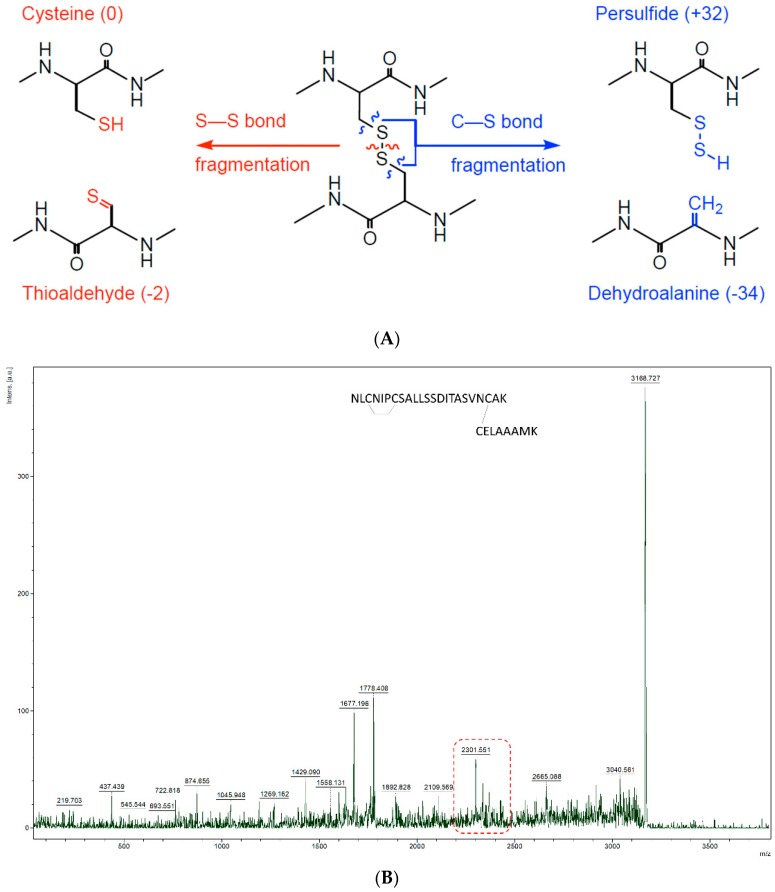

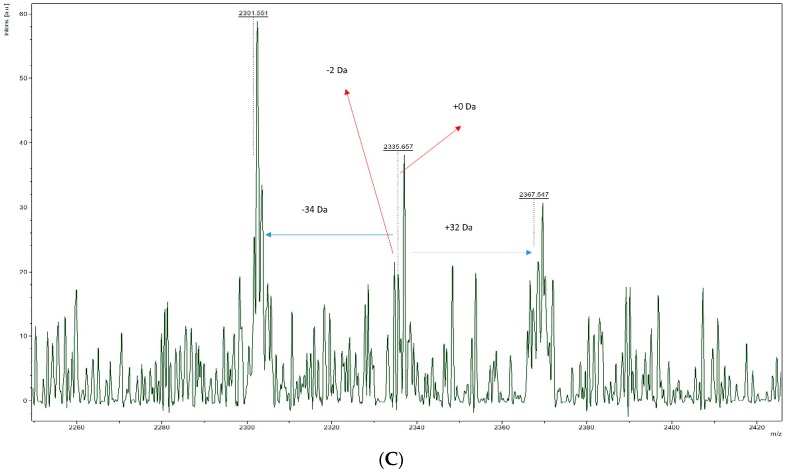
(**A**) Schematic representation of characteristic DSB fragment ions. (**B**) MS/MS spectrum of peptide NLCNIPCSALLSSDITASVNCAK—CELAAAMK, at *m*/*z* 3168.479. (**C**) Zooming in to the red box of MS/MS spectrum of 3168.479 delivers a characteristic diagnostic triplet at the inter-link peptide NLCNIPCSALLSSDITASVNCAK confirming the presence of a disulphide bond.

With the ultimate goal of easy characterization of intact disulphide bonds, we dug deeper in the potential of our HCCA:aniline mixture. Important to note is that, as currently agreed upon in literature, no sequence-related proof can be found from inter-peptide DSBs via MALDI, as cyclic intra-peptide DSBs are very hard to ionize. Therefore, we focused on the detection of inter-peptide DSBs in Lysozyme C digest. Though many DSBs could be fragmented by the use of HCCA matrix alone, some of them could only be isolated and fragmented in the HCCA-ani matrix. Interestingly, those peptides only fragmented by HCCA-ani were more complex. For example, the *m*/*z* 3168.479 peak, is potentially a two peptide [NLCNIPCSALLSSDITASVNCAK and CELAAAMK] linkage containing both an inter- and intra-DSB cross-link. Although no sequence information can be obtained from intra-peptide DSB via MALDI-TOF MS/MS, inter-peptide DSB’s should, if the crosslink is abundant enough, provide sequence information. However, an easier way is to search for diagnostic MS/MS ions that are specific for DSBs. In the case of peak *m*/*z* 3168.479, the existence of the inter-chain crosslink was confirmed by the presence of diagnostic DSB fragment ion peak, with a mass difference of −34 Da, −2 Da and +32 Da, around peptide NLCNIPCSALLSSDITASVNCAK, one of the inter-linked peptides in the MS/MS spectrum (Figure 3B,C). These diagnostic ions, previously termed as the characteristic ‘disulphide triplet marker ions’ [1] represent fragment ions which correspond to breakages through, and on, either side of the DSB where the crosslinked peptides take place in (Figure 3A). Although complete sequence information of this complicated peptide linkage could not be obtained, by adding aniline, which improves the characterisation of more complex linkages, we obtained fragmentation evidence of the inter-chain DSB. With this critical information we were able to confirm the existence of the DSB that would have otherwise been missed with a traditional MALDI matrix preparation. Therefore, we can say that the use of aniline/HCCA matrix for MALDI-TOF/TOF MS measurements of non-reduced proteins provides proof of existence of complex DSB peptides in these samples. We therefore encourage the use of aniline in MALDI matrices when intact disulphide bonded peptides need to be analysed.

### 3.6. Confirmation of the Inter-Peptide Disulphide Bonds Characterisation Results in a More Complex Sample

**Figure 4:**
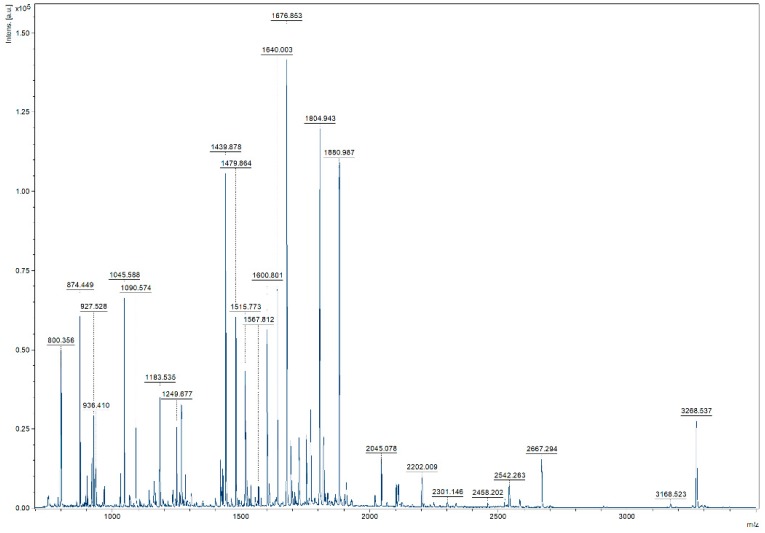
MALDI-MS spectrum of complex peptide digest measured with HCCA:aniline as matrix.

**Figure 5:**
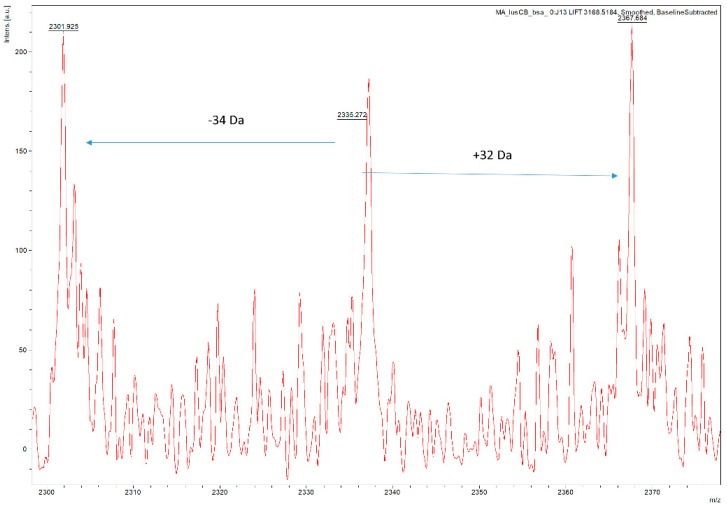
Zoomed-in *m*/*z* region of MALDI MS/MS spectrum focussing on the NLCNIPCSALLSSDITASVNCAK peptide in the DSB linkage. The existence of the inter-chain crosslink was confirmed by the presence of diagnostic DSB fragment ion peaks, with a mass difference of −34 Da and +32 Da, corresponding to the dehydroalanine and persulphide form of the peptide after DSB breakage.

To confirm the potential of our HCCA:aniline MALDI matrix in DSB characterization, we increased the complexity of the lysozyme C digest by adding two other protein digests (beta-lactoglobulin and BSA) to the sample. Comparison of the MS spectrum of this mixed protein sample (Figure 4), to the lysozyme C digest MS spectrum (Figure 2), enables to see an increase in complexity as significantly more *m*/*z* values are detected. To confirm that our methodology also works in this more complex sample, we isolated and fragmented the same disulphide bond-containing peptide as before. Figure 5 illustrates the fragmentation spectrum of *m*/*z* 3168.51, the peak which is potentially a two peptide [NLCNIPCSALLSSDITASVNCAK and CELAAAMK] linkage. In addition, we searched for proof in the region of interest confirming the existence of the disulphide bond, as the characteristic marker ions, specific for the longest of the two peptides in the crosslink, should be present there. In this zoomed-in region, the existence of the inter-chain crosslink was confirmed by the presence of diagnostic DSB fragment ion peaks, with a mass difference of −34 Da and +32 Da, corresponding to the dehydroalanine and persulphide form of the peptide after DSB link breakage. Therefore, we can say that in more complex peptide digests, our approach is still valuable.

## 4. Conclusions

DHAP matrix allows good detection of non-reduced DSB peptides via MALDI TOF/TOF MS but currently has a suboptimal fragmentation efficiency for these peptides, certainly compared to HCCA as MALDI matrix. The addition of aniline to HCCA improves the ability to fragment the potential disulphide linked precursor ions, regardless of their complexity. These preliminary results thus demonstrate that the use of aniline/HCCA matrix for MALDI-TOF/TOF MS measurements of non-reduced proteins provides proof of existence of complex DSB peptides in these samples.

## Figures and Tables

**Table 1:** Overview of MALDI signal intensities (arbitrary units) of the *m*/*z* values of the peptides in the calibration mixture and the different matrix: additives combinations. The ‘/’ means the peaks is absent in the spectrum.

Matrix-Additive	757.399	1046.542	1296.685	1347.735	1619.822	2093.086	2465.198	3147.471
HCCA-m	3176	529,648	266,182	217,525	31,248	348,344	5511	156
HCCA-sc	/	/	/	/	/	/	/	/
HCCA-aa	1881	163,495	108,986	3199	9359	9359	688	/
HCCA-ANI	/	511,570	558,446	492,022	367,807	367,807	105,862	994
DHAP-m	/	212,573	207,126	47,283	87,757	87,757	7605	385
DHAP-sc	5094	61,582	153,401	28,359	149,998	149,998	1450	431
DHAP-aa	/	203,525	389,998	100,072	213,742	213,742	14,655	909
DHAP-ANI	/	45,705	63,629	15,356	73,780	73,780	9816	212

**Table 2:** Overview of the *m*/*z* values of the potential DSB peptides in the lysozyme C digest measured with different matrix: additives combinations. Spectra were collected with HCCA or DHAP as MALDI matrix (m) and three different additives ammonium acetate (aa), sodium citrate (sc) and aniline (ani). The “x” means the peak is present in the MALDI spectrum, “/” means the peaks is absent in the spectrum. If the x is bold, a MS/MS spectrum of that *m*/*z* value could be obtained. * indicates a native inter-peptide disulphide bond, all other are scrambled bonds.

	497.214	582.242	667.270	1083.49	1168.520 *	1183.470	1268.498	1515.701 *	1600.729	1669.770	1769.748	1869.726	2101.979	2201.957	2534.188	2582.201	2667.229	3168.479	3268.457 *	3600.688	4667.188
HCCA-m	/	/	/	/	/	x	**x**	**x**	**x**	/	**x**	**x**	**x**	**x**	x	/	**x**	/	**x**	/	/
HCCA-sc	/	/	/	/	/	/	/	/	/	/	/	/	/	/	/	/	/	/	/	/	/
HCCA-aa	/	/	/	**x**	**x**	x	x	**x**	**x**	/	**x**	**x**	/	/	/	/	**x**	/	**x**	/	/
HCCA-ANI	/	/	/	**x**	**x**	x	**x**	**x**	**x**	**x**	**x**	**x**	**x**	**x**	**x**	**x**	**x**	**x**	**x**	x	/
DHAP-m	/	/	/	x	x	x	x	x	x	/	x	x	x	**x**	x	/	x	/	**x**	x	/
DHAP-sc	/	/	/	/	/	/	**x**	**x**	x	/	/	x	x	**x**	x	/	x	/	**x**	x	/
DHAP-aa	/	/	/	x	x	x	**x**	x	x	/	/	x	x	**x**	x	/	x	/	**x**	x	/
DHAP-ANI	/	/	/	**x**	x	x	x	x	x	/	/	x	x	**x**	x	/	x	x	**x**	**x**	/

## Supplementary Materials

The following are available online at http://www.mdpi.com/2409-9279/1/3/33/s1, Figure S1: Supplementary Figure S1A displays the signal intensities of Somatostatin 28 measured with different MALDI HCCA matrix:aniline ratios. In Supplementary Figure S1B, the HCCA-matrix:aniline ratio comparison for the Lysozyme C digest is displayed.
