# Supplementary material for: Improved Detection and Fragmentation of Disulphide-Linked Peptides
Source: Methods Protoc. 2018 Sep 3;1(3):33. doi: 10.3390/mps1030033 (PMC6481087; doi:10.3390/mps1030033)
Supplement: Supplementary file 1 [file mps-01-00033-s001.pdf]

A)

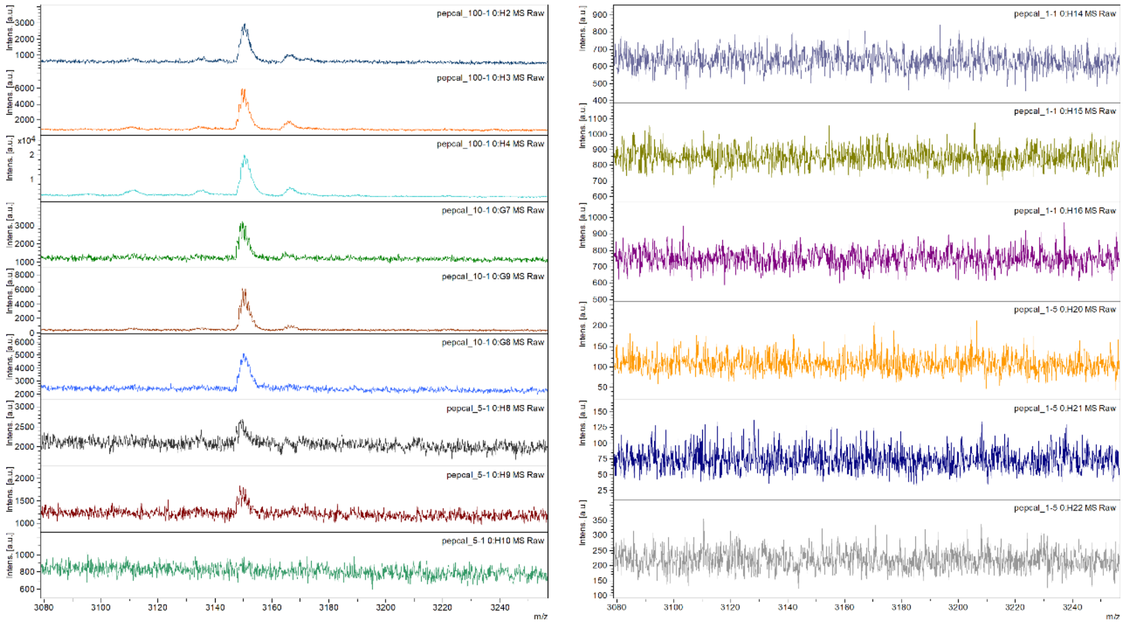


B)

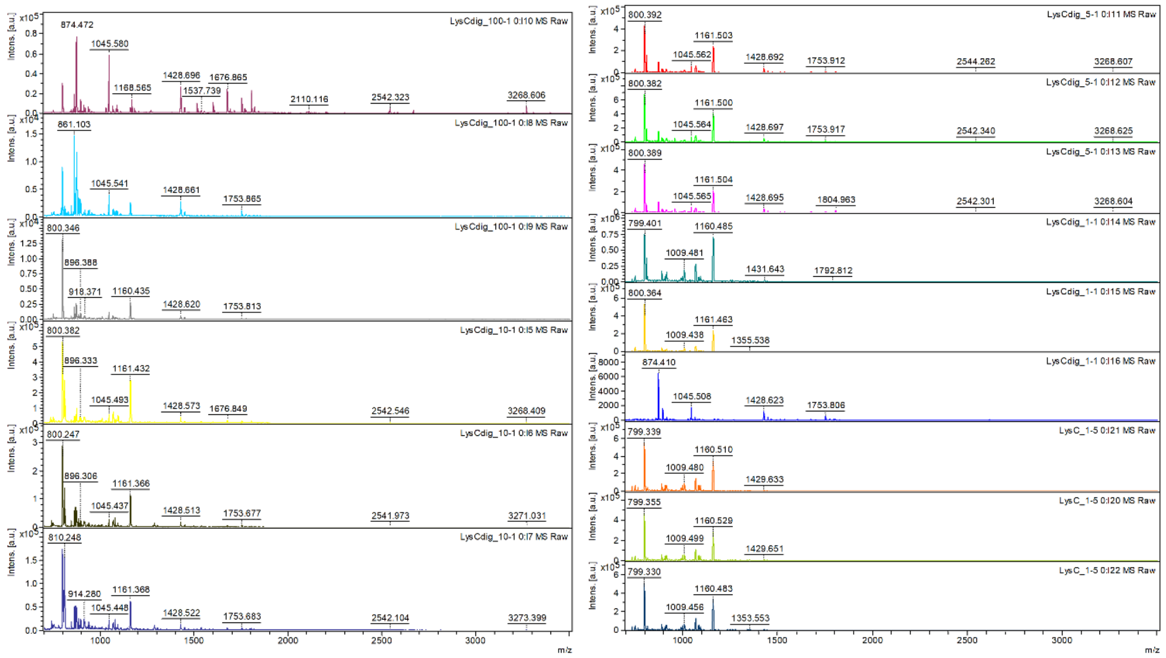


**Figure S1.** Supplementary figure S1A displays the signal intensities of Somatostatin 28 measured with different MALDI HCCA matrix:aniline ratios. In supplementary figure S1B, the HCCA-matrix:aniline ratio comparison for the Lysozyme C digest is displayed.
